# Coronary arterovenous fistula: to treat or not to treat?

**DOI:** 10.1186/s13019-015-0256-3

**Published:** 2015-04-07

**Authors:** Federica Jiritano, Filippo Prestipino, Pasquale Mastroroberto, Massimo Chello

**Affiliations:** 1 Cardiac Surgery Unit, University Campus Bio-Medico of Rome, Rome, 00128 Italy; 2 Cardiac Surgery Unit, “Magna Graecia” University of Catanzaro, Catanzaro, 88100 Italy

**Keywords:** Coronary artery disease, Congenital heart defect, Coronary circulation

## Abstract

We reported the case of a 68-year old male with chest pain. The coronary angiography showed the disease of the left anterior descending coronary artery and, incidentally, an arteriovenous coronary fistula between this coronary branch and the pulmonary artery. The patient underwent off-pump coronary bypass through a left mini thoracotomy. In the present case, after a series of detailed exams, we decided not to close the fistula for several reasons, but mainly because of the singular localization of an atherosclerotic plaque proximal to the origin of the fistula. Therefore, under specific conditions, it may not always be mandatory to close the coronary arteriovenous fistulas.

## Background

Coronary arteriovenous fistulas (CAF) are rare vascular communications between a coronary artery and venous structures such as, right sided chambers, pulmonary artery, coronary sinuses, and superior vena cava [1]. They are present in 0,002% of the general population and represent 0,13% of congenital coronary anomalies [1]. The most common site of drainage is the right ventricle (41%), while drainage of the CAF into the pulmonary trunk has been reported in 17% of cases [2]. They are associated with other cardiac disease such as coronary atherosclerosis, and therefore may enhance myocardial ischemia [2]. We report the case of a CAF found incidentally during coronary angiography in a patient evaluated for acute myocardial infarction, who underwent coronary artery bypass graft (CABG) surgery.

## Case presentation

**Figure 1:**
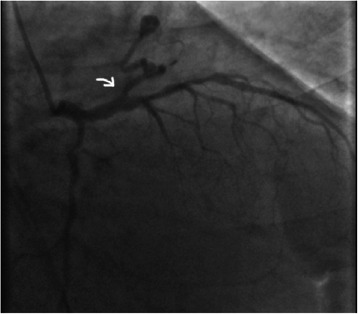
**CAF is indicated by the white arrow.**

A 68-year-old male was admitted because of chest pain and dyspnea (NYHA class IV). He was obese and with a past medical history of diabetes mellitus, hypertension, hyperlipidemia, smoking and cerebral ischemic event. Physical examination was unremarkable. Electrocardiography showed a normal sinus rhythm, Q waves in the anterior leads (suggesting anterior myocardial infarction). Trans-thoracic echocardiography showed hypokinesia of the anterior wall, but the overall systolic function of the left ventricle is preserved (EF = 55%). Coronary angiogram revealed significant stenosis on the left descending coronary artery (LAD), while the left circumflex artery (LCX) and the right coronary artery (RCA) were not significantly diseased. Moreover, there was a small fistula connection arising from the LAD, just before a proximal stenosis, and draining into the main pulmonary artery (Figure 1). Scrupulous pre-operative diagnostic tests allowed determining that the CAF was not hemodynamically relevant. After the coronary angiography (which allowed to find out the CAF) the patient underwent cardiac catheterization to assess whether the CAF created or not a significant left-to-right shunt. The exam showed a small left-to-right shunt, normal pulmonary resistance and normal pulmonary to systemic ratio (Qp/Qs). Furthermore, a trans esophageal echocardiography (TEE) was performed to better estimate the fistula. No unexpected or unusual structure was observed. The exam did not revealed any enlarged coronary artery and the right cardiac chambers were not dilated. The results of all these tests have established that the fistula had small caliber and was not significant from the hemodynamic point of view. Consequently, further additional studies did not seem useful. In consideration of the preoperative studies, which revealed that the fistula was not relevant, the cardiac surgeon decided not to treat it. Thereafter, the surgical team, in agreement with the patient, chose a type of minimally invasive surgical approach. The patient underwent conventional off pump CABG x 1 (Left internal thoracic artery to LAD) in left mini-thoracotomy. Because of its small size and the particular origin-site of the CAF, before a significant proximally stenosis of the LAD, it was not considered necessary to close it. Transit time flowmetry of LIMA-LAD graft was 45 ml/min, with IP: 1.0. The intra and post-operative course was smooth. Patient was discharged home on the 5^th^ post-operative day. At six month follow-up, the patient was asymptomatic and in stable condition, and the ergometric test was negative for myocardial ischemia.

## Conclusions

Coronary arterovenous fistulas (CAF) are uncommon congenital anomalies of the coronary arteries or more rarely acquired secondary to cardiac interventions or cardiac surgery [1]. CAF may arise from any coronary arteries, although more often originate from RCA and LDA. LCX is rarely involved. In the most cases, CAF drain into venous structures such as, right sided chambers, pulmonary artery, coronary sinuses, and superior vena cava [1]. The majority of the adult patients remain asymptomatic according to size and localization of the CAF, and the coronary flow reserve [2]. Liberthson et al. reported that younger patients (under 20 years) are more often asymptomatic. Other side, patients older than 20 years old are symptomatic for dyspnoea on exertion (35%), fatigue (8%) or angina (22%) [2]. Coronary angiography remains the most common method of diagnosis of CAF. The natural history of the CAF is variable. Spontaneous closure secondary to spontaneous thrombosis, although uncommon, has been reported [3]. The management is debated, and recommendations are based on small retrospective series or few case reports [2, 4]. However, general agreement exists that symptomatic patients should be treated, but is still controversial in patients without symptoms. The choice between trans-catheter closure (TCC) of CAF and surgical intervention is still controversial. TCC, which started in 1983, may be applied in favourable anatomy conditions and when surgery is not possible [5]. Surgical closure of CAF was first performed by Bjork and Crafoord in 1947, and it is still the most common employed technique [6]. Ligation of the CAF may be achieved with or without cardiopulmonary bypass, when there is a simple and easily accessible fistula [5]. In the present case, the authors preferred to do not treat the CAF because: - the small size of the fistula did not make it hemodynamically relevant; the origin-site of the CAF, immediately before the proximally significant stenosis on the LAD, does not allow the fistula to steal blood flow from the coronary bypass conduit.

A detailed preoperative study allowed us to evaluate best the CAF. In conclusion, a careful preoperative assessment can reveal the presence of a not significant CAF in terms of hemodynamic, and therefore, that may not require any treatment.

## Consent

Written informed consent was obtained from the patient for publication of this Case report and any accompanying images. A copy of the written consent is available for review by the Editor-in-Chief of this journal.

## Abbreviations

CAF: Coronary arteriovenous fistulas
CABG: Coronary artery bypass graft
LAD: Left descending coronary artery
LCX: Left circumflex artery
RCA: Right coronary artery
TEE: Trans esophageal echocardiography
TCC: Trans-catheter closure
